# Supplementary material for: Optimizing the Ion Conductivity and Mechanical Stability of Polymer Electrolyte Membranes Designed for Use in Lithium Ion Batteries: Combining Imidazolium-Containing Poly(ionic liquids) and Poly(propylene carbonate)
Source: Int J Mol Sci. 2024 Jan 27;25(3):1595. doi: 10.3390/ijms25031595 (PMC10855450; doi:10.3390/ijms25031595)
Supplement: Supplementary file 1 [file ijms-25-01595-s001.zip › ijms-2813749-supplementary.pdf]

Supporting Information

Optimizing the Ion Conductivity and Mechanical Stability of Polymer Electrolyte Membranes Designed for Use in Lithium Ion Batteries: Combining Imidazolium-Containing Poly(ionic liquids) and Poly(propylene carbonate)

Nataliya Kiriy [1,*], Sezer Özenler [1], Pauline Voigt [1], Oliver Kobsch [1], Jochen Meier-Haack [1], Kerstin Arnhold [1], Andreas Janke [1], Upenyu L. Muza [1], Martin Geisler [1,2], Albena Lederer [1,2], Doris Pospiech [1], Anton Kiriy [3,*] and Brigitte Voit [1,4]

1 Leibniz-Institut für Polymerforschung Dresden e.V., Hohe Str. 6, 01069 Dresden, Germany;

2 Department Chemistry and Polymer Science, Stellenbosch University, Matieland 7600, South Africa;

3 beeOLED GmbH, Niedersedlitzer Strasse 75c, 01257 Dresden, Germany

4 Organische Chemie der Polymere, Technische Universität Dresden, 01062 Dresden, Germany

*Correspondence: kiriy-nataliya@ipfdd.de (N.K.); anton.kiriy@beeoled.com (A.K.); Tel.: +49-4658497 (N.K.)

This supporting information contains 26 figures, 6 tables and 1 reference over 25 pages.

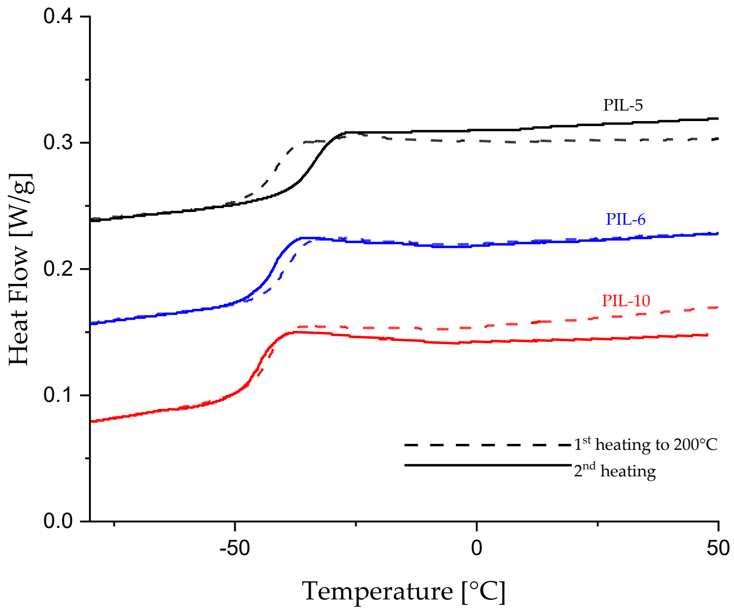

Figure S1. DSC curves of PILs for Tg derive, given in Table 1: PIL-5 (black color); PIL-6 (blue); PIL-10 (red color), where dotted and solid curves are 1st, 2nd heating respectively.

Table S1. The thicknesses and measured resistance of pure PILs: PIL-5, PIL-6; PIL-10 used for the calculation of the ion conductivity given in Table 1 (corresponding Nyquist plots are presented in Figure S2) at RT **.

Dry samples	Thickness* [cm]	Resistance [kΩ]
PIL-5	0.1	18.5
PIL-6	0.1	13
PIL-10	0.1	6.8

*the thickness of Teflon ring (Figure S25a) was used as a thickness of the samples.

** room temperature (RT)

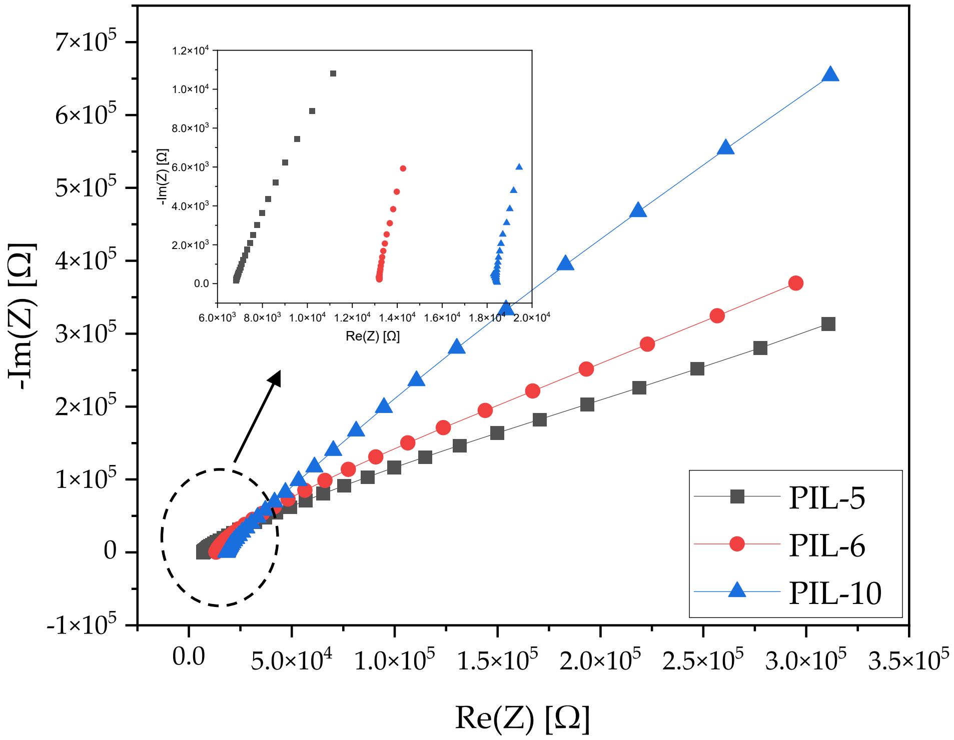

Figure S2. Nyquist plots of the pure PILs for resistance derive, given in Table S1: PIL-5 (black color); PIL-6 (red color); PIL-10 (blue color) at RT.

Table S2. The weights, the calculated thicknesses, according to the equation 2 and the resistances of PIL-6 and bicomponent mixture of PIL-6 with 10, 20, 30 wt% LiTFSI at RT and 60 °C used for the calculation of ion conductivity (according to equation 1) given in Table 2 (corresponding Nyquist plots are presented in Figures S3, S4).

Samples	Weight [mg]	Thickness [cm]	Resistance at RT [kΩ]	Resistance at 60°C [kΩ]
PIL-6	22	0.039	4.698	0.574
PIL-6 with 10 wt% LiTFSI	21	0.038	9.406	1.495
PIL-6 with 20 wt% LiTFSI	20	0.036	55.24	3.279
PIL-6 with 30 wt% LiTFSI	21	0.038	99.86	4.709

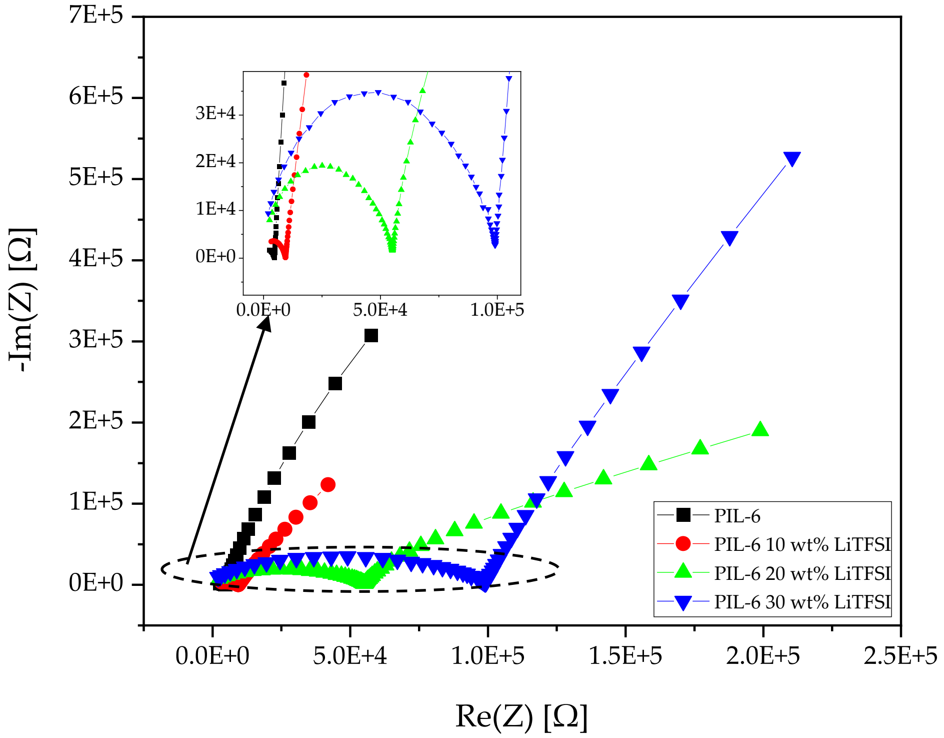

Figure S3. Nyquist plots of PIL-6 (black color) and its mixtures with LiTFSI: 10 wt% (red color), 20 wt% (green color), 30 wt% (blue color) at RT for resistance derive, given in Table S2.

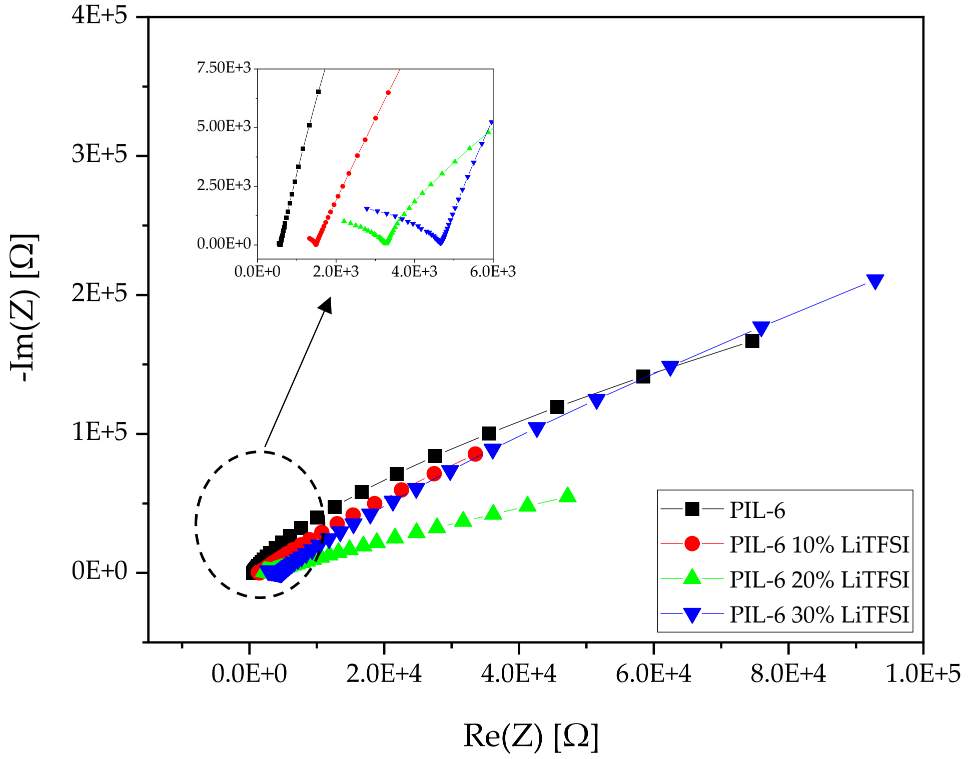

Figure S4. Nyquist plots of PIL-6 (black color) and its mixtures with LiTFSI: 10 wt% (red color), 20 wt% (green color), 30 wt% (blue color) at 60 °C for resistance derive, given in Table S2.

Table S3. The thicknesses and the resistances of bicomponent and tricomponent membranes of PPC/LiTFSI and PPC/PILs/LiTFSI in 1/0.3 wt/wt and 1/1/0.6 wt/wt/wt ratio respectively, used for the calculation of ion conductivity (according to equation 1) given in Table 3 (corresponding Nyquist plots are presented in Figures S5, S6).

Samples	Thickness* [cm]	Resistance at RT [kΩ]
PPC/PIL-5/ LiTFSI	0.044	31.10
PPC/PIL-6/LITFSI	0.044	23.530
PPC/PIL-10/LITFSI	0.044	16.820
PPC/ LiTFSI	0.060	8102

* The thicknesses were measured by digital caliper gauge.

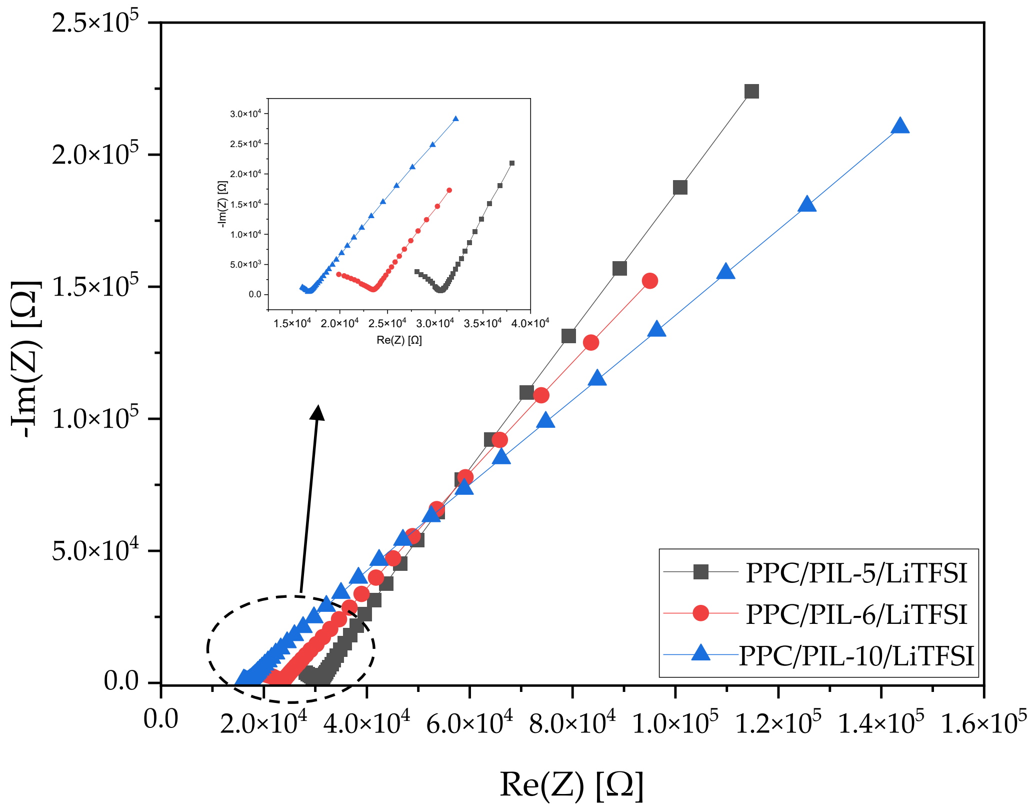

Figure S5. Nyquist plots of tricomponent membranes of PPC/PILs/ LiTFSI (1/1/0.6 wt/wt/wt) with: PIL-5 (black color); PIL-6 (red color); PIL-10 (blue color) on separator Celgard 2500 at RT for resistance derive, given in Table S3.

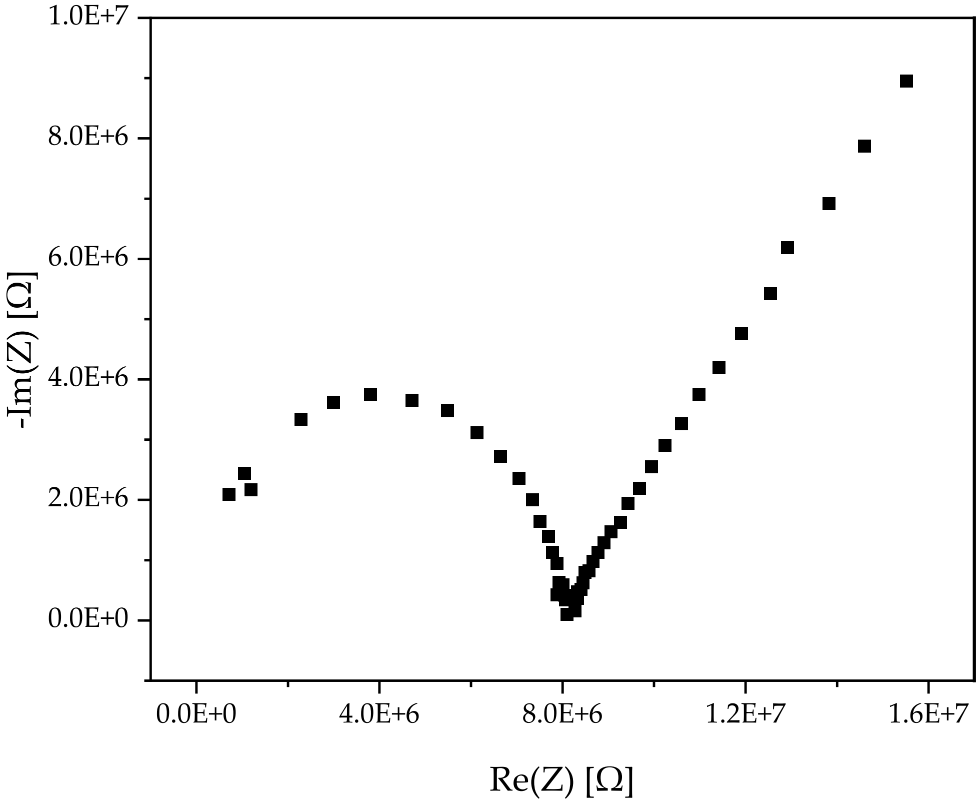

Figure S6. Nyquist plots of bicomponent membrane of the PPC/LiTFSI 1/0.3 wt/wt at RT on separator Celgard 2500 at RT for resistance derive, given in Table S3.

Table S4. The resistance and calculated ion conductivity of tricomponent membrane PPC/ PIL-6/LiTFSI in various wt/wt/wt at RT (corresponding Nyquist plots are presented in Figures S7a, S7b).

PPC/ PIL-6/LiTFSI	Resistance [kΩ]	Thickness* [cm]	Ionic conductivity [$S \cdot cm^{-1}$]
3/1/0.4	277	0.047	$2x10^{-7}$
3/1/0.8	121	0.05	$5x10^{-7}$
3/1/1.2	40	0.07	$2\ x10^{-6}$
1/1/0.2	90	0.05	$7x10^{-7}$
1/1/0.4	61.5	0.05	$1x10^{-6}$
1/1/0.6	15	0.07	$5\ x10^{-6}$

* The thicknesses were measured by digital caliper gauge.

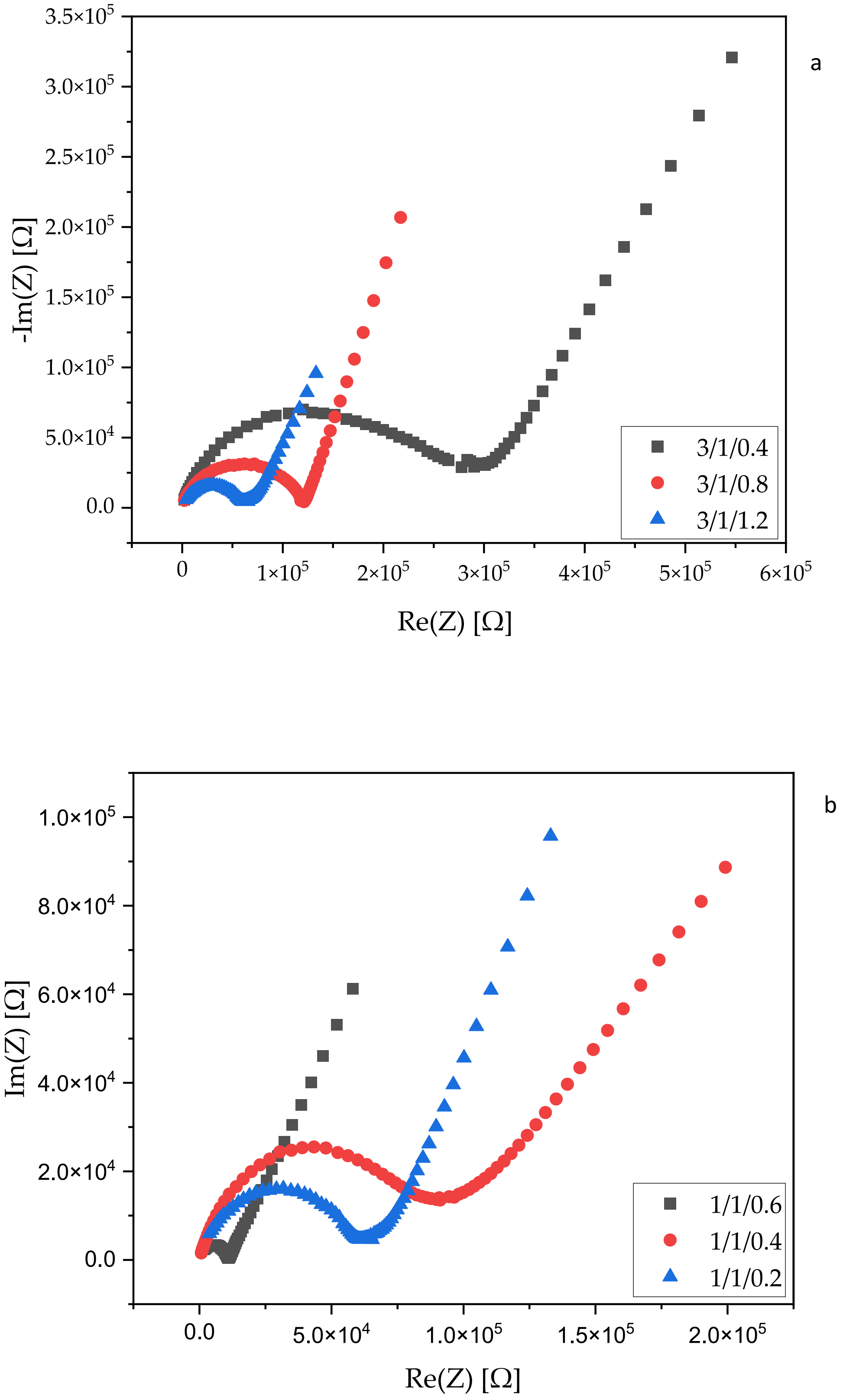

Figure S7. Nyquist plots of the tricomponent membranes PPC/PIL-6/LiTFSI in various wt/wt/wt ratio of components at RT: a) in 3/1/1.2 (blue color); 3/1/0.8 (red color); 3/1/0.4 (black

color); b) 1/1/0.6 (black color); 1/1/0.4 (red color); 1/1/0.2 (blue color) for resistance derive, given in Table S4.

Table S5. The thicknesses and measured resistance of the bicomponent PPC/LiTFSI (1/0.3 wt/wt) and tricomponent PPC/PIL-6/LiTFSI (3/1/1.2 wt/wt/wt) and PPC/PIL-10/LiTFSI (1/1/0.6 wt/wt/wt) membranes used for the calculation of the conductivity given in Table 4 (corresponding Nyquist plots are presented in Figure S8, S9).

Entry	Membrane	Amount of acetonitrile [wt%]	Thickness* [cm]	Resistance at RT [kΩ]
#1	PPC/LiTFSI	10	0.038	27.44
#2	PPC/LiTFSI	20	0.051	6.4
#3	PPC/LiTFSI	30	0.05	0.062
#4	PPC/PIL-10/LiTFSI	2	0.068	86.6
#5**	PPC/PIL-6/LiTFSI	8	0.07	0.26
#6	PPC/PIL-10/LiTFSI	30	0.044	0.020

* The thicknesses were measured by digital caliper gauge.

**The Nyquist plot is given in Figure S10 blue color.

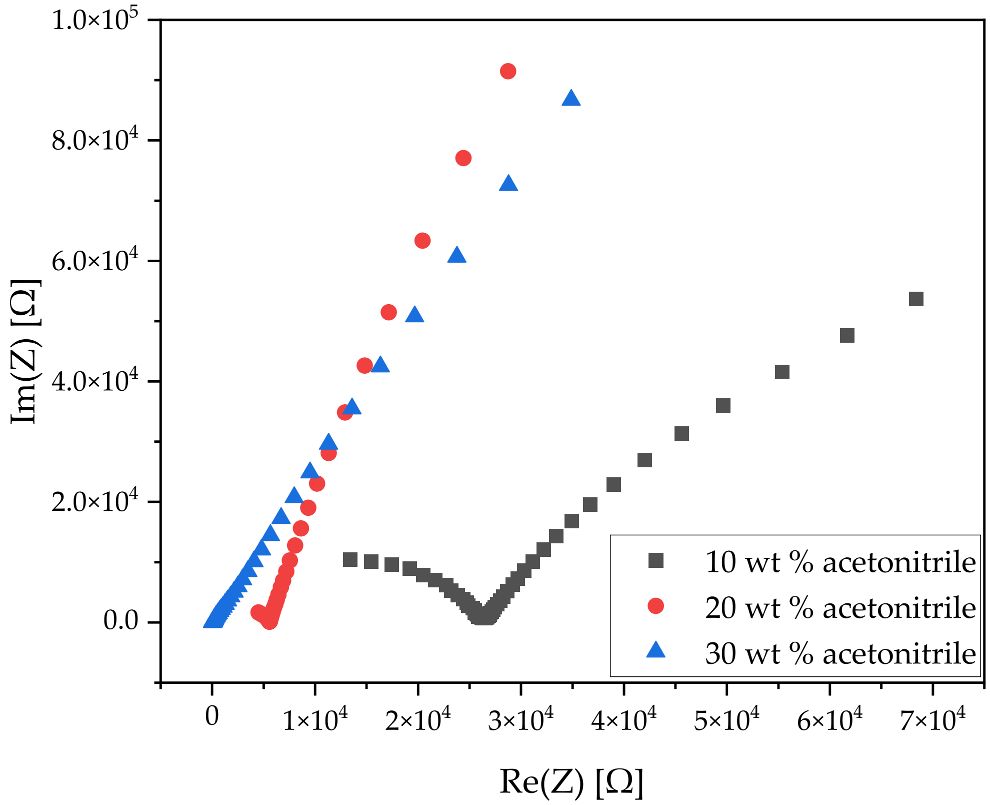

Figure S8. Nyquist plots of the bicomponent membranes PPC/LiTFSI (1/0.3 wt/wt) at RT: with 10 wt% (black color), 20 wt% (red color), 30 wt% (blue color) of acetonitrile for resistance derive, given in Table S5 for entry #1- #3.

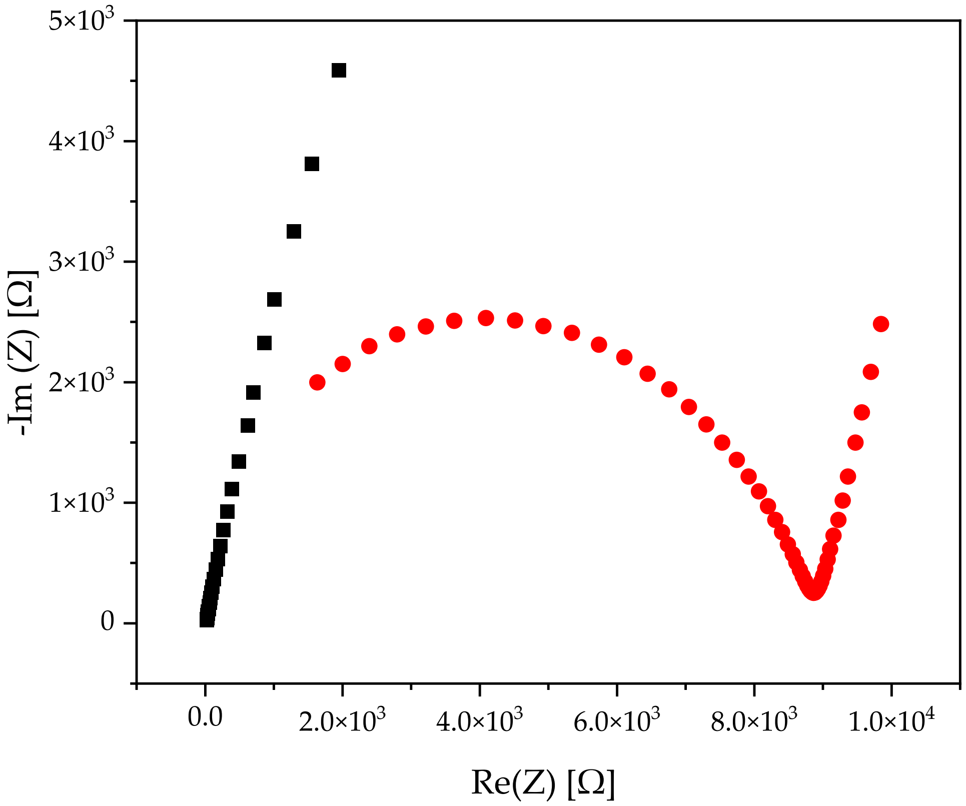

Figure S9. Nyquist plots of the tricomponent membrane PPC/PIL-10/LiTFSI (1/1/0.6 wt/wt/wt): dry (red curve) and containing 30 wt% acetonitrile (black curve) at RT for resistance derive, given in Table S5 for entry #6.

Table S6. The resistance and calculated ion conductivity tricomponent membranes of PPC/PIL-6/LiTFSI with different wt/wt/wt ratio with different wt% of acetonitrile at RT (corresponding Nyquist plots are presented in Figure S10).

Entry	PPC/PIL/LiTFSI	Amount of acetonitrile [wt%]	Resistance, [kΩ]	Thickness* [cm]	Ionic conductivity [S·cm^{-1}]
#1	3/1/0.4	15	0.27	0.047	2x10^{-4}
#2	3/1/0.8	13	0.14	0.05	4x10^{-4}
#3	3/1/1.2	8	0.26	0.07	3x10^{-4}

* The thicknesses were measured by digital caliper gauge

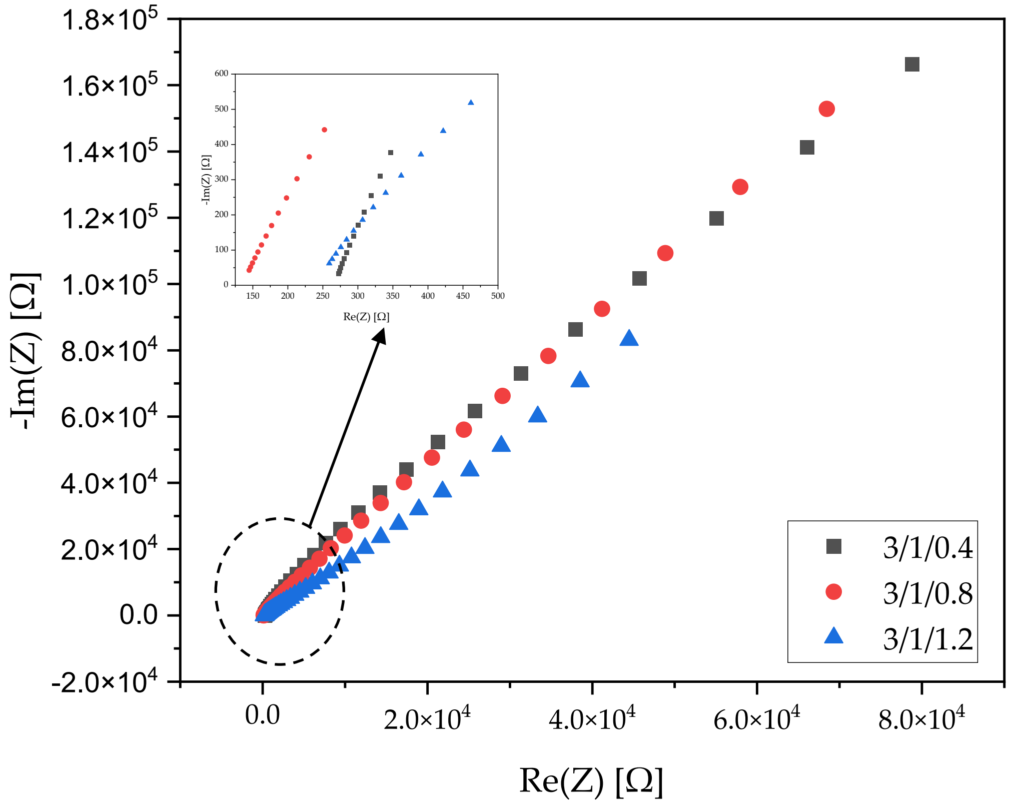

Figure S10. Nyquist plot of the tricomponent membranes PPC/PIL-6/LiTFSI in various wt/wt/wt ratio of components at RT: 3/1/0.4 (black color); 3/1/0.8 (red color); 3/1/1.2 (blue color), after exposure in acetonitrile vapor for 0,5 hours for resistance derive, given in Table S6.

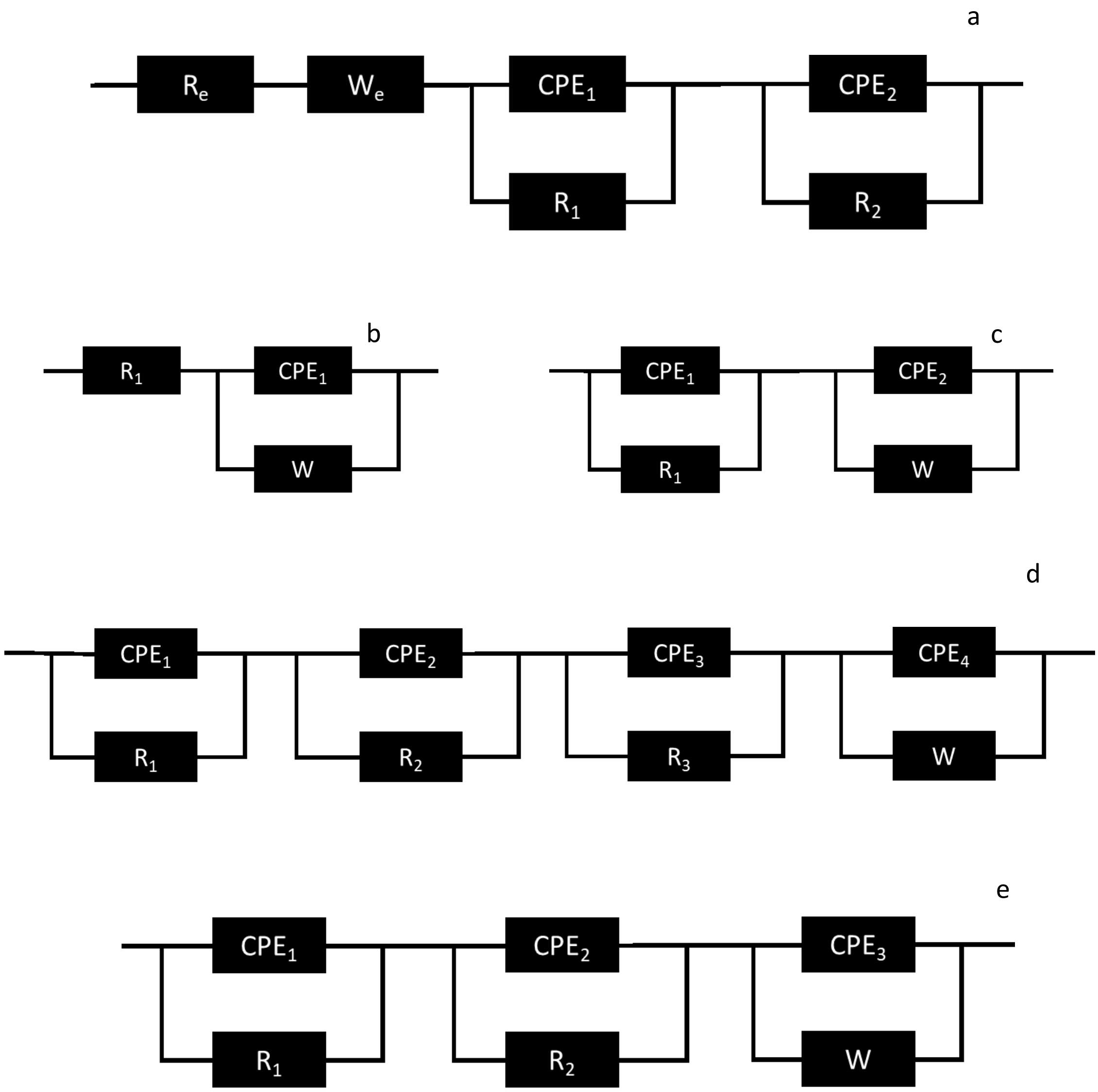

Figure S11. Equivalent circuits used as fitting model for Nyquist plots: (a) in Figure 3; (b) in Figures S2, S8 (for membranes PPC/LiTFSI in 1/0.3 wt/wt with 20 and 30wt% acetonitrile), S9 (for membrane PPC/PIL-10/LiTFSI in 1/1/0.6 wt/wt/wt with 30wt% acetonitrile); (c) in Figures S3-S6, S9(for membrane PPC/PIL-10/LiTFSI in 1/1/0.6 wt/wt/wt dry); (d) in Figure S7a and S7b; (e) in Figure S10.

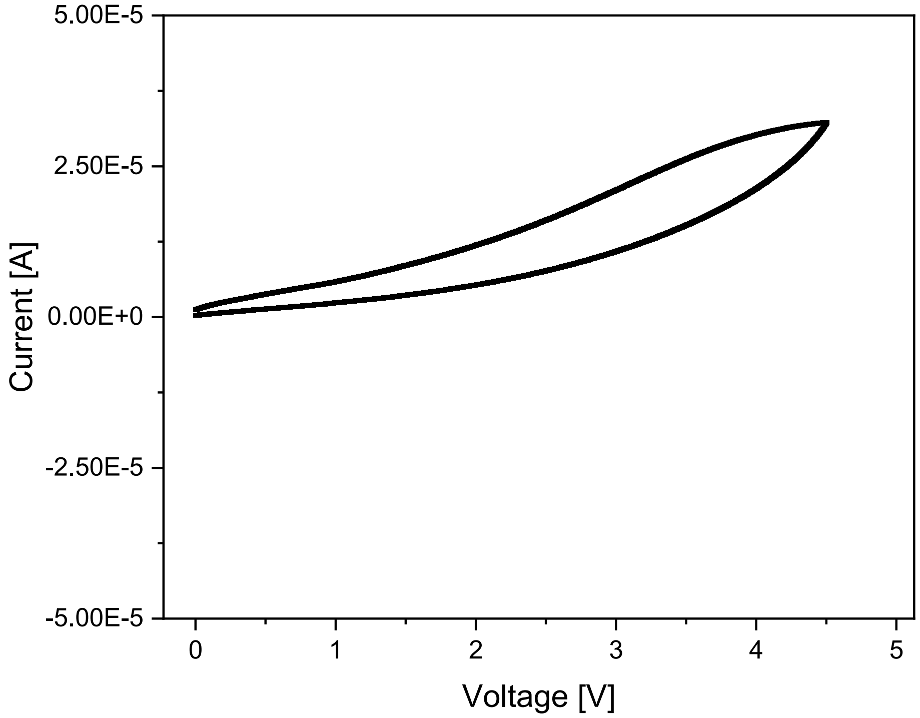

Figure S12. The typical CV diagram of the PPC/PIL-10/LiTFSI 1/1/0.6 wt/wt/wt vs. Li/Li+ in a Swagelok cell at RT, scan rate 100 mV/s.

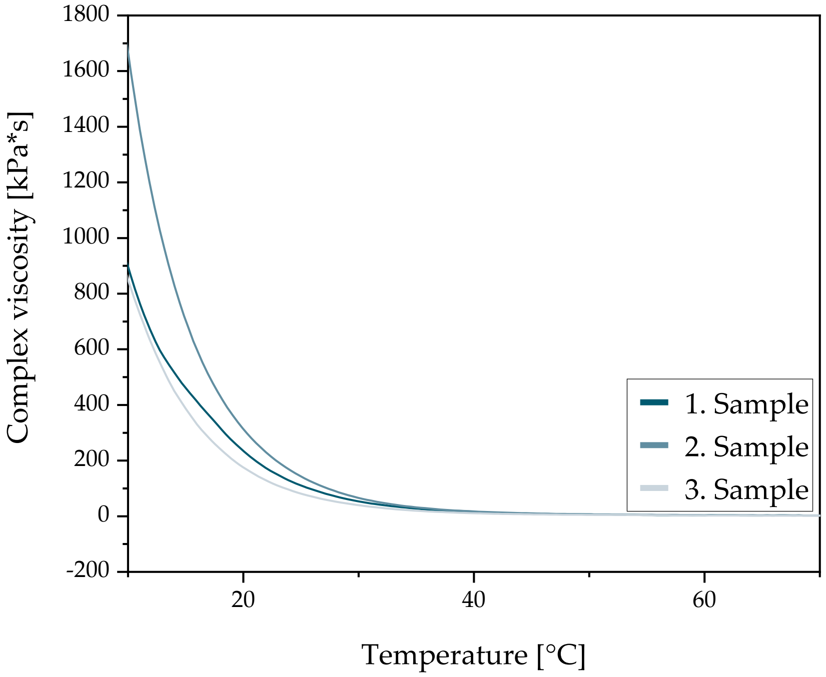

Figure S13. The viscosity dependence from temperature of tricomponent membrane PPC/PIL-10/ LiTFSI 1/1/0.6 wt/wt/wt, for viscosity derive at 20 °C and 60 °C, given in Table 6.

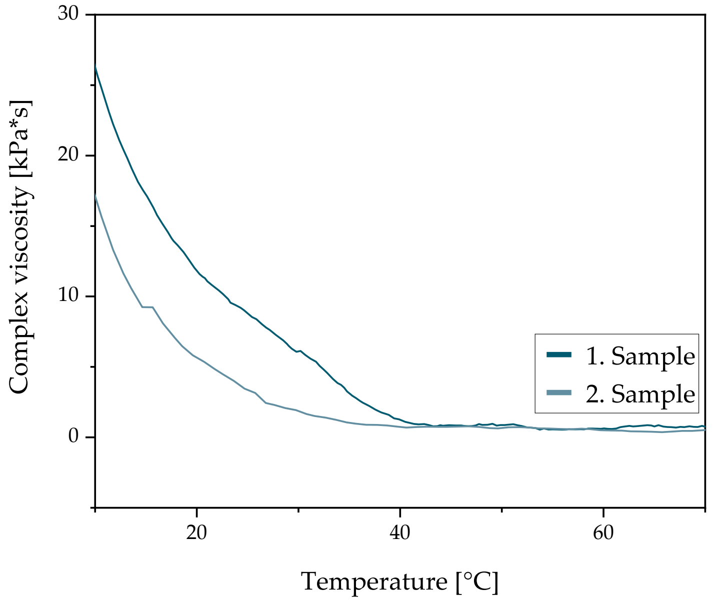

Figure S14. The viscosity dependence from temperature of bicomponent membrane PIL-10/PPC (1/1 wt/wt), for viscosity derive at 20 °C and 60 °C, given in Table 6.

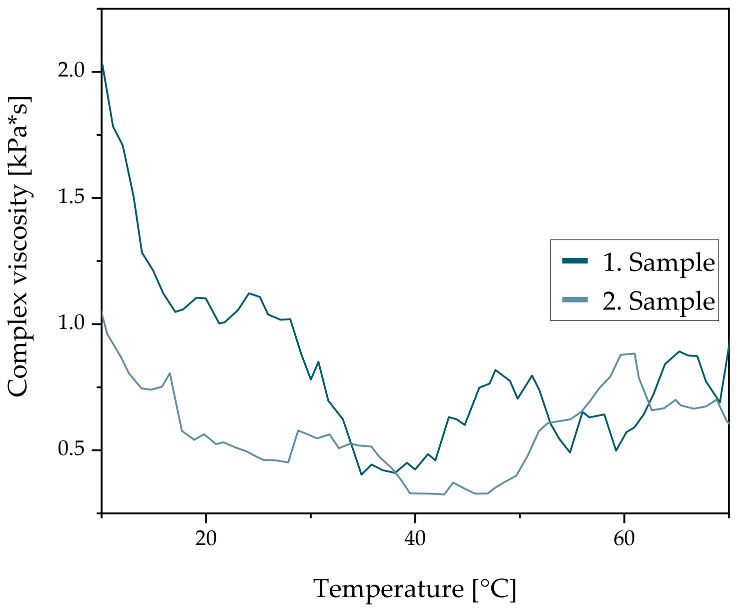

Figure S15. The viscosity dependence from temperature of PIL-10, for viscosity derive at 20 °C and 60 °C, given in Table 6.

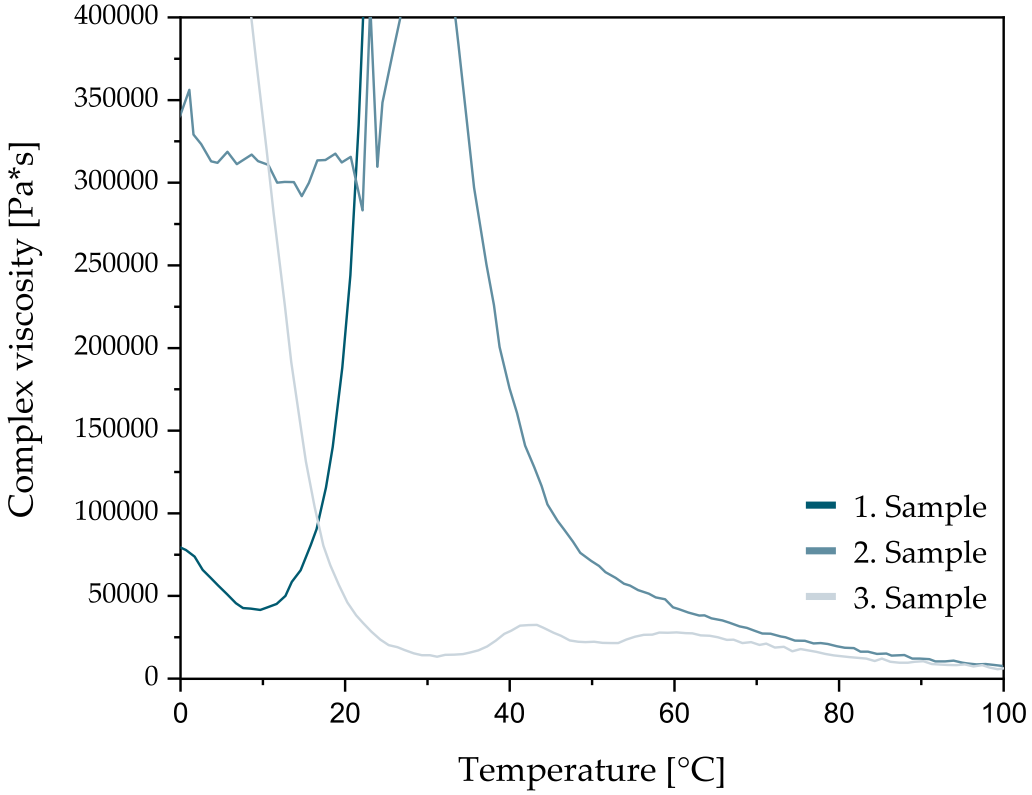

Figure S16. The viscosity dependence from temperature of PPC, for viscosity derive at 20 °C and 60 °C, given in Table 6.

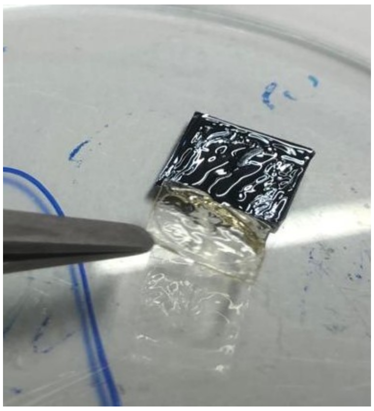

Figure S17. Photograph illustrates the process of the removal of the PPC film from the matrix of PPC /PIL-10 (1/1 wt/wt) on Si wafer.

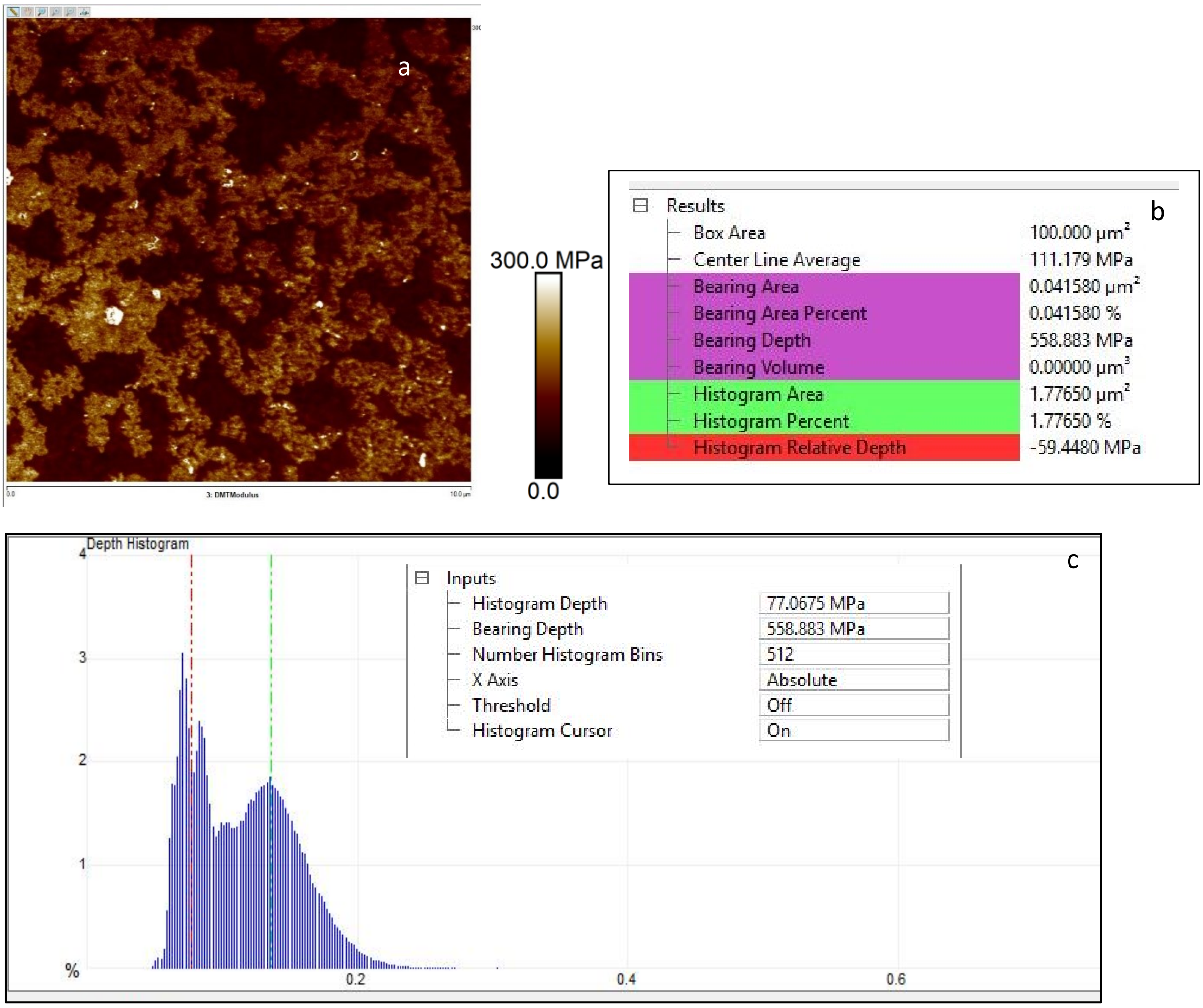

Figure S18. AFM Quantitative nanomechanical analysis of the tricomponent membrane PPC/PIL-10/LiTFSI in 1/1/0.6 wt/wt/wt: (a) DMT Modulus image; (b) Modulus histogram analysis; (c) Modulus histogram.

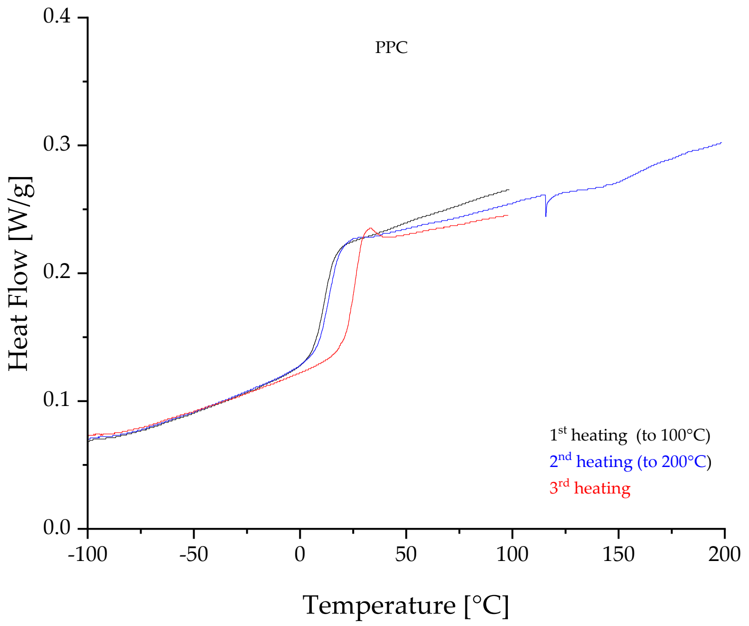

Figure S19. DSC curve of the pure PPC: black, blue, red color are 1st, 2nd, 3rd heating respectively, for Tg derive given in Table 8.

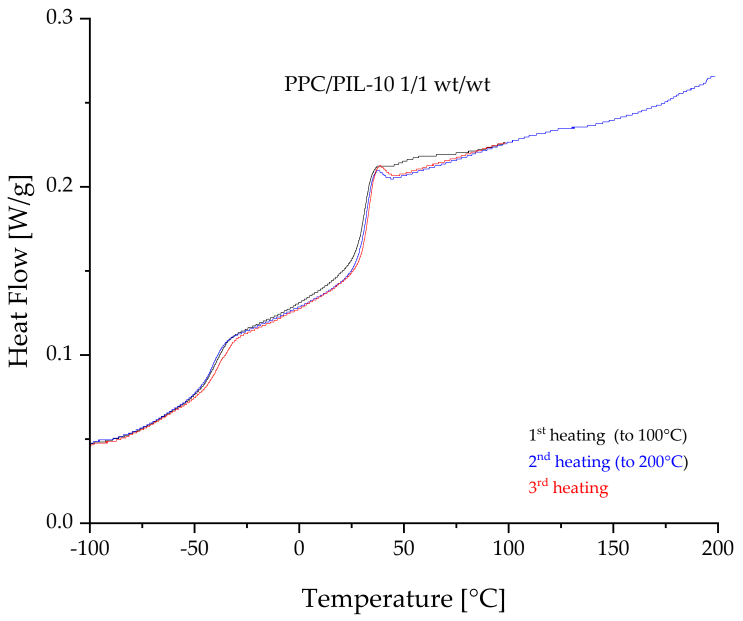

Figure S20. DSC of the bicomponent membrane PPC and PIL-10 1/1 wt/wt: black, blue, red color are 1st, 2nd, 3rd heating respectively, for Tg derive given in Table 8.

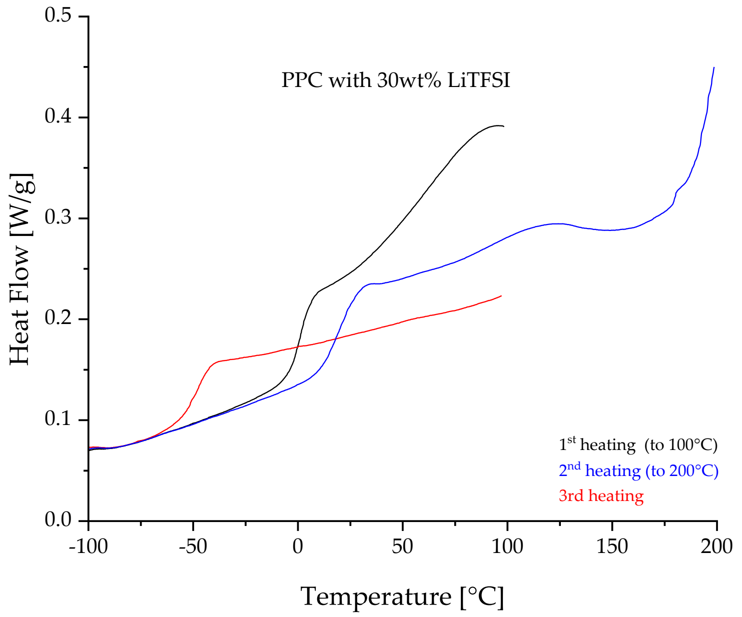

Figure S21. DSC curves of bicomponent membrane PPC and 30wt% LiTFSI: black, blue, red color are 1st, 2nd, 3rd heating respectively, for Tg derive given in Table 8.

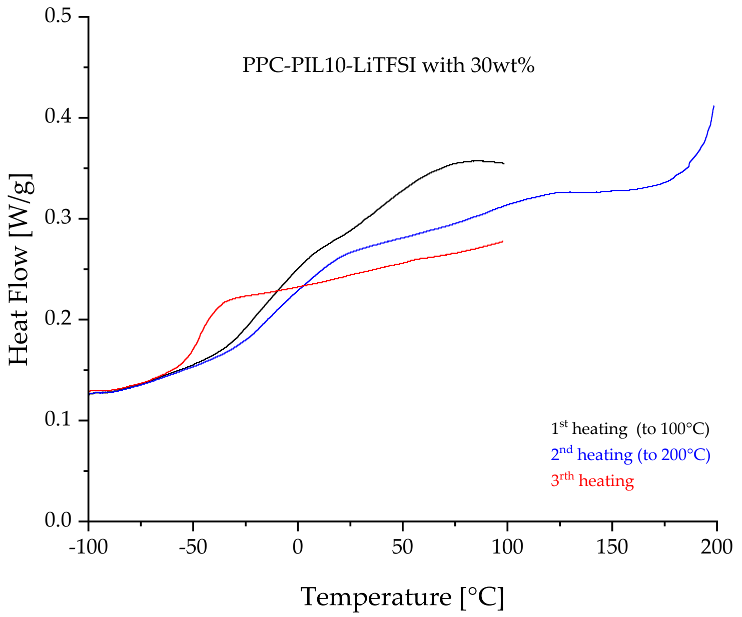

Figure S22. DSC curves of tricomponent membrane PPC/PIL-10/LiTFSI 1/1/0.6: black, blue, red color are 1st, 2nd, 3rd heating respectively, for Tg derive given in Table 8.

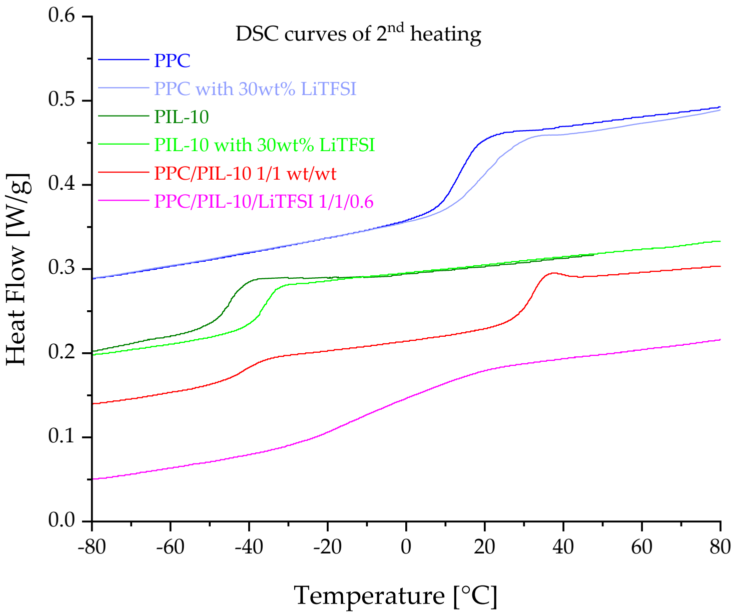

Figure S23: Summary of DSC curves of 2nd heating of all components for Tg derive, given in Table 8: PPC (dark blue color); bicomponent membrane PPC with LiTFSI 30 wt% (light blue color); PIL-10 (dark green color); PIL-10 with 30wt% LiTFSI (light green color); bicomponet membrane PPC/PIL-10 1/1 wt/wt (red color); tricomponent membrane PPC/PIL-10/ LiTFSI 1/1/0.6 wt/wt/wt (magenta color).

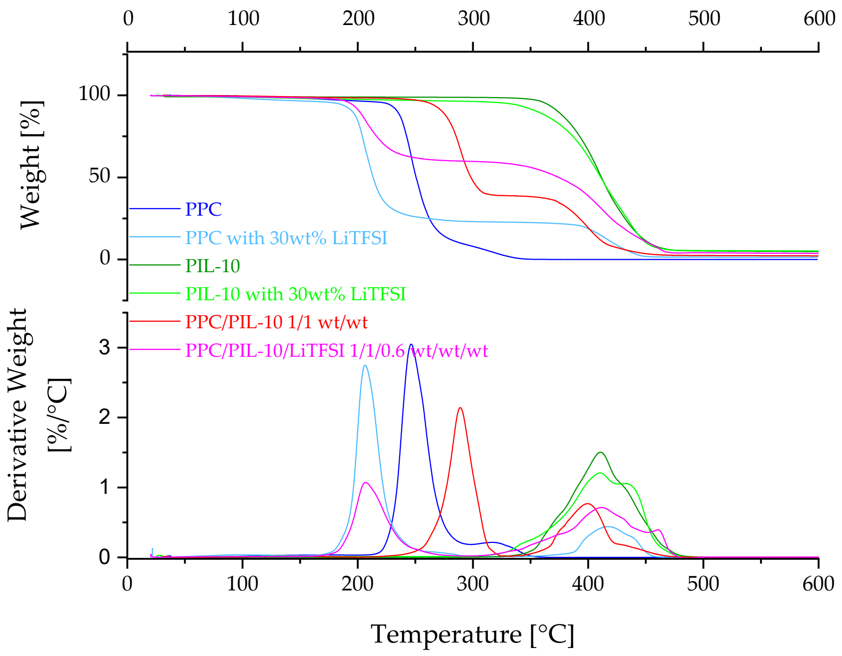

Figure S24. TGA curves for $T_{start\ of\ dec.}$ and $T_{max\ of\ dec}$ derive, given in Table 8: pure PPC (dark blue color); bicomponent membrane PPC with 30wt% LiTFSI (light blue color); PIL-10 (dark green color); PIL-10 with LiTFSi_30 wt% (light green color); bicomponent membrane PPC/PIL-10 1/1 wt/wt (red color); tricomponent membrane PPC/PIL10/LiTFSI 1/1/0.6 (magenta color).

1. Synthesis of Compound 1

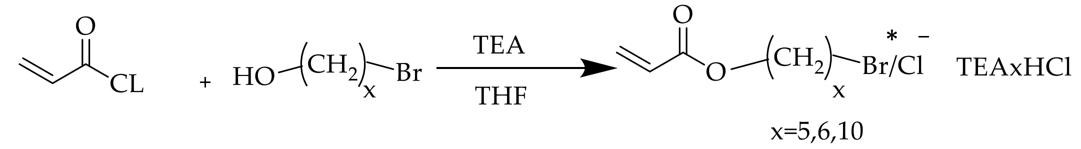

2. Synthesis of Compound 2

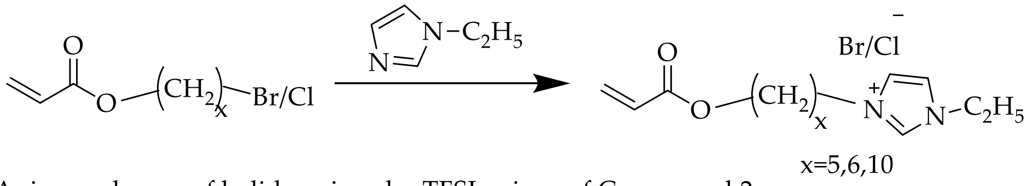

3. Anion exchange of halide anions by TFSI anions of Compound 2

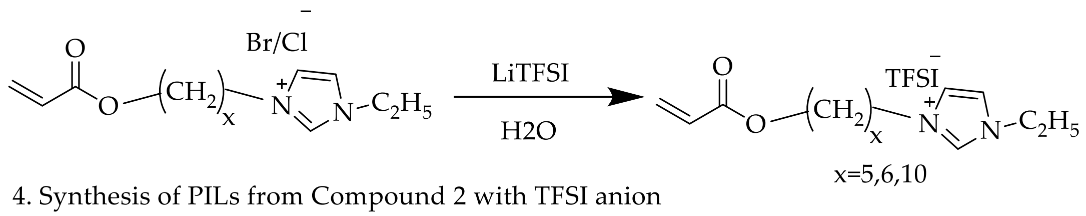

4. Synthesis of PILs from Compound 2 with TFSI anion

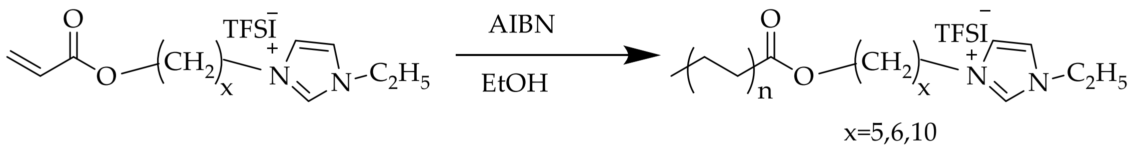

Figure S25. 4 steps procedure of synthesis of PILs.

* The product of the first step contains the mixture of Compound 2 with bromo- and chlor anions that was indicated by the NMR analysis [1].

NMR Data of the Compound 2 (product of step 3).

With X=5 *^{1}H NMR (500 MHz, DMSO-d$_6$) δ_H ppm* 1.30 (quin, *J*=7.30 Hz, 2 H) 1.43 (t, *J*=7.41 Hz, 3 H) 1.63 (quin, *J*=7.01 Hz, 2 H) 1.83 (quin, *J*=7.50 Hz, 2 H) 4.10 (t, *J*=6.62 Hz, 2 H) 4.15 (t, *J*=7.57 Hz, 2 H) 4.19 (q, *J*=7.30 Hz, 2 H) 5.93 (dd, *J*=10.25, 1.42 Hz, 1 H) 6.16 (dd, *J*=17.30, 10.20 Hz, 1 H) 6.31 (dd, *J*=17.18, 1.42 Hz, 1 H) 7.79 (d, *J*=8.20 Hz, 2 H) 9.17 (s, 1 H)

With X=6 *^{1}H NMR (500 MHz, DMSO-d$_6$) δ_H ppm* 1.28 (quin, *J*=7.30 Hz, 2 H) 1.36 (quin, *J*=7.30 Hz, 2 H) 1.43 (t, *J*=7.41 Hz, 3 H) 1.62 (quin, *J*=7.01 Hz, 2 H) 1.81 (quin, *J*=7.50 Hz, 2 H) 4.10 (t, *J*=6.62 Hz, 2 H) 4.15 (t, *J*=7.57 Hz, 2 H) 4.19 (q, *J*=7.30 Hz, 2 H) 5.93 (dd, *J*=10.25, 1.42 Hz, 1 H) 6.16 (dd, *J*=17.30, 10.20 Hz, 1 H) 6.31 (dd, *J*=17.18, 1.42 Hz, 1 H) 7.79 (d, *J*=8.20 Hz, 2 H) 9.17 (s, 1 H)

With X=10 *^{1}H NMR (500 MHz, DMSO-d$_6$) δ_H ppm* 1.28 (quin, *J*=7.30 Hz, 2 H) 1.36 (quin, *J*=7.30 Hz, 2 H) 1.43 (t, *J*=7.41 Hz, 3 H) 1.62 (quin, *J*=7.01 Hz, 2 H) 1.81 (quin, *J*=7.50 Hz, 2

H) 4.10 (t, *J*=6.62 Hz, 2 H) 4.15 (t, *J*=7.57 Hz, 2 H) 4.19 (q, *J*=7.30 Hz, 2 H) 5.93 (dd, *J*=10.25, 1.42 Hz, 1 H) 6.16 (dd, *J*=17.30, 10.20 Hz, 1 H) 6.31 (dd, *J*=17.18, 1.42 Hz, 1 H) 7.79 (d, *J*=8.20 Hz, 2 H) 9.17 (s, 1 H)

NMR Data of PILs

PIL-5 *^{1}H NMR (500 MHz, DMSO-d$_6$) δ_H ppm* 1.27 (bs, 2 H) 1.42 (t, *J*=7.41 Hz, 3 H) 1.55 (bs, 2 H) 1.78 (bs, 2 H), 3,93 (bs, 2H) 4.13 (bs, 2 H) 4.19 (q, *J*=7.57 Hz, 2 H) 4.20 (q, *J*=7.30 Hz, 2 H) 7.73 (bs, 1 H) 7.79 (s, 1 H) 9.13 (bs, 1 H)

PIL-6 *^{1}H NMR (500 MHz, DMSO-d$_6$) δ_H ppm* 1.27 (bs, 4 H) 1.42 (t, *J*=7.41 Hz, 3 H) 1.52 (bs, 2 H) 1.78 (bs, 2 H), 3,93 (bs, 2H) 4.13 (bs, 2 H) 4.19 (q, *J*=7.57 Hz, 2 H) 4.20 (q, *J*=7.30 Hz, 2 H) 7.73 (bs, 1 H) 7.79 (s, 1 H) 9.13 (bs, 1 H)

PIL-10 *^{1}H NMR (500 MHz, DMSO-d$_6$) δ_H ppm* 1.24 (bs, 12 H) 1.42 (t, *J*=7.41 Hz, 3 H) 1.52 (bs, 2 H) 1.78 (bs, 2 H), 3,93 (bs, 2H) 4.13 (bs, 2 H) 4.19 (q, *J*=7.57 Hz, 2 H) 4.20 (q, *J*=7.30 Hz, 2 H) 7.73 (bs, 1 H) 7.79 (s, 1 H) 9.13 (bs, 1 H)

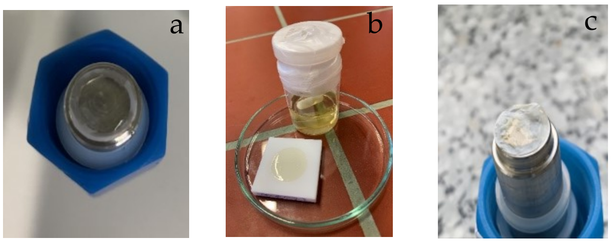

Figure S26. Photograph of samples: a) pure PIL inside Teflon ring; b) acetonitrile solution in glass vial and tricomponent membrane on Teflon mold prepared from PPC/PILs /LiTFSI 1/1/0.6 wt/wt/wt; c) tricomponent membrane on separator Celgard 2500.

Reference

1. Röchow, ET.; Coeler, M.; Pospiech, D.; Kobsch, O.; Mechtaeva, E.; Vogel, R.; Voit, B.; Nikolowski, K.; Wolter, M. In Situ Preparation of Crosslinked Polymer Electrolytes for Lithium Ion Batteries: A Comparison of Monomer Systems. *Polymers* **2020**, *12*, 1707.
